# Supplementary material for: Reconciling Mining with the Conservation of Cave Biodiversity: A Quantitative Baseline to Help Establish Conservation Priorities
Source: PLoS One. 2016 Dec 20;11(12):e0168348. doi: 10.1371/journal.pone.0168348 (PMC5173368; doi:10.1371/journal.pone.0168348)
Supplement: S1 Dataset — (ZIP) [file pone.0168348.s002.zip › Taxa/Serra Sul/SS_2010/S11D_64.pdf]

S11D-64		1ª	AB	2ª	AB	ZON
Annelida						
Clitellata						
Oligochaeta	jovens	5	0,00348			P
Arthropoda						
Arachnida						
Acari						
Ixodida						
Argasidae						
Ornithodoros	sp.			1		E
Parasitiformes						
Mesostigmata	sp.1	1				P
	sp.2			3		P A
	sp.8	1				P
	sp.9	1				P
Laelapidae	sp.1	1				P
	sp.3	1				E
Ologamasidae	sp.1	1				P
Sarcoptiformes						
Oribatida	sp.2			1		P
Acaridae	sp.1			1		E
Trombidiformes						
Tydeoidea						
Cunaxidae	sp.2	1		1		P
Labdostomatidae	sp.1	1				P
Scutacaridae	sp.1			1		P
	sp.1	7		4		P A
	sp.2	5				E P A
	sp.7			1		P
Amblypygi						
Charinidae	jovens	5	0,00348	3	0,0041	P A
Charinus	sp.	48	0,03343	40	0,0552	A
	sp.2	4	0,00279	4	0,0055	P A
Phrynidae						
Heterophrynus	sp.	8	0,00557	7	0,0097	P
	sp.1	1	0,0007			E
Araneae	jovens	23	0,01602	1	0,0014	P
Araneidae						
Alpaida	*septemmammata*	1				E
Corinnidae	jovens	3	0,00209	1	0,0014	P
Ctenidae	jovens			2	0,0028	P
Mimetidae	jovens	1				E
Ochyroceratidae	jovens	1		3		P
	sp.1	3		3		P A
Ochyrocera	sp.1	14		7		E P A
Speocera	sp.1	2				P
Oonopidae	jovens	1		1		P
gr. *Xycarphius*	sp.2	1				P
Pholcidae	jovens	1		3		E P
Ninetinae	sp.1	1		2		E P
Salticidae	jovens	1				E
Amphidraus	sp.1	1				E
Scytodidae	jovens	5		4		E P
Scytodes	*eleonorae*	3	0,00209	1	0,0014	P
	globula	2	0,00139			E
	sp.	12	0,00836	17	0,0235	P
Segestriidae	jovens	3				E P
Ariadna	sp.1	1		1		P
Tetrablemmidae	jovens	2		1		P A
Matta	sp.1	4		4		E P A
Theraphosidae	jovens	5	0,00348	3	0,0041	E P
aff. *Holothele*	sp.1	1	0,0007			P
gen.2	sp.1			1	0,0014	E

Theridiosomatidae		jovens		1		1		P
Opiliones				51	0,03552	41	0,0566	
Cyphophthalmi								
Neogoveidae								
	Canga		*renatae*	3		2		P A
Laniatores								
Escadabiidae			sp.1	12		5		P A
Stygnidae		jovens		2	0,00208	1	0,0014	P A
			sp.1	3	0,00209	2	0,0028	E P
Palpigradi								
Eukoeneniidae								
	Allokoenenia		sp.1	1				P
Pseudoscorpiones								
Bochicidae		jovens				2		P
			sp.1	4		4		P A
Chernetidae		jovens		2				E
	Spelaeochernes		sp.1	1		3		P
Chthoniidae								
Chthoniidae			sp.1	4		1		P A
	Pseudochthonius		sp.1	5		3		P
Olpiidae			sp.1	2		2		E P
Chilopoda		jovens		60	0,04178			P
Pleurostigmophora								
Geophilomorpha								
Geophilidae			sp.1	7	0,00487	2	0,0028	E P
Diplopoda		jovens		34	0,02368	1	0,0014	E
Polydesmida								
Fuhrmannodesmidae			sp.1	1				P
			sp.3	2		2		P A
			sp.4			1		A
Pyrgodesmidae			sp.2	5	0,00348	5	0,0069	P A
Polyxenida								
Hypogexenidae			sp.1			1		P
Entognatha								
Diplura								
Campodeidae			sp.1	1		1		P
Insecta								
Blattodea		jovens		68	0,04735			P
Blaberidae		jovens		3	0,00209	2	0,0028	P A
			sp.1	1	0,0007			P
Blattidae		jovens		1	0,0007			P
			sp.3	1	0,0007			E
Coleoptera				1				P
Collembola								
Arthropleona								
Entomobryoidea		jovens						
Cyphoderidae			sp.1			2		A
Paronellidae			sp.1			1		P
			sp.4	3		3		P A
Symphypleona								
Sminthuroidea			sp.1	1				P
			sp.2	8		4		E P
Diptera		jovens		5		8		E P A
Brachycera								
Camillidae			sp.	1				P
Drosophilidae								
	Drosophila		*eleonore*	4				E P A
Streblidae								
	Trichobius		sp.	3				P
Nematocera								
Cecidomyiidae								
	Cecidomyiinae		sp.	1				P
Chironomidae			sp.			4		P A

Psychodidae						
Lutzomyia	*longipalpis*	1				E
Pericoma	sp.	5		1		E P A
Phlebotominae	sp.			1		P
Pintomyia	*gruta*	1				E
Sciopemyia	*sordellii*	9		1		E P A
Sciaridae						
Bradysia	sp.			1		P
Hemiptera						
Heteroptera						
aff. Pyrrhocoroidea						
Cydnidae	jovens	2				P
Cydninae	sp.1	12		3		E P A
Homoptera	jovens	51	0,03552			
Cixiidae	jovens	2		1		E P
	sp.7	1	0,0007			A
Hymenoptera	jovens			1		P
Vespoidea						
Formicidae						
Camponotus	*atriceps*	9	0,00627	7	0,0097	E P
	sp.1	1				P
Cephalotes	sp.1	1				E
Gnamptogenys	*striatula*	1		1		E P
Hypoponera	sp.1			1		P
Odontomachus	*bauri*	1		2		P
Pachycondyla	*striata*	4		2		P
Pheidole	sp.1	1				E
	sp.2	1				E
Solenopsis	sp.1	13		6		E P A
Wasmania	*auropunctata*	1	0,0007			P
Isoptera	sp.	2				E P
Termitidae						
Nasutitermes	sp.	6		7		E P
Lepidoptera	jovens	3		3		E P
Cossoidea						
Limacodidae	sp.1	1	0,0007			E
Noctuoidea						
Noctuidae	sp.2			1	0,0014	E
Tineoidea	sp.1	2		1		E P
Neuroptera						
Myrmeleontidae	jovens	1				E
Orthoptera						
Ensifera	sp.2			1	0,0014	P
Phalangopsidae						
Paraclodes	sp.1			4	0,0055	P
Phalangopsis	sp.1	###	0,69638	554	0,7652	E P A
Psocoptera						
Psocomorpha	jovens			1		P
Trogiomorpha						
Lepidopsocidae						
Loxopholia	sp.1			1		E
Psyllipsocidae	jovens			1		P
Thysanura	jovens	1				P
Ateluridae	sp.1			1		E
Nicoletiidae	jovens	1		1		E P
	sp.1	4		2		P A
Malacostraca						
Isopoda						
Dubioniscidae	sp.1	1				E
Philosciidae	sp.1	7		2		P
Scleropactidae	sp.			2		E P
Chordata						
Amphibia						

Anura							
Neobatrachia							
Strabomantidae							
Pristimantis	*fenestratus*			11	0,0152	P
Mammalia							
Chiroptera							
Emballonuridae							
Peropteryx	*kappleri*	1	0,0007			
	sp.			2	0,0028	P
Phyllostomidae	sp.			10	0,0138	A
Carollia	*perspicillata*	6	0,00418			
Glossophaga	*soricina*	5	0,00348			
Lonchophylla	cf. *dekeyseri*	2	0,00139			
	thomasi	1	0,0007			
Phyllostomus	*latifolius*	1	0,0007			
Mollusca							
Gastropoda							
Subulinidae							
Lamellaxis	sp.	2				P
